# G-Channel Restoration for RWB CFA with Double-Exposed W Channel

**DOI:** 10.3390/s17020293

**Published:** 2017-02-05

**Authors:** Chulhee Park, Ki Sun Song, Moon Gi Kang

**Affiliations:** Yonsei University, Deptartment of Electrical and Electronic Engineering, 50 Yonsei-ro, Seodaemun-gu, Seoul 03722, Korea; ascaron5@gmail.com (C.P.); iamhoh@naver.com (K.S.S.)

**Keywords:** G-channel restoration, red–white–blue color filter array, multispectral image sensor

## Abstract

In this paper, we propose a green (G)-channel restoration for a red–white–blue (RWB) color filter array (CFA) image sensor using the dual sampling technique. By using white (W) pixels instead of G pixels, the RWB CFA provides high-sensitivity imaging and an improved signal-to-noise ratio compared to the Bayer CFA. However, owing to this high sensitivity, the W pixel values become rapidly over-saturated before the red–blue (RB) pixel values reach the appropriate levels. Because the missing G color information included in the W channel cannot be restored with a saturated W, multiple captures with dual sampling are necessary to solve this early W-pixel saturation problem. Each W pixel has a different exposure time when compared to those of the R and B pixels, because the W pixels are double-exposed. Therefore, a RWB-to-RGB color conversion method is required in order to restore the G color information, using a double-exposed W channel. The proposed G-channel restoration algorithm restores G color information from the W channel by considering the energy difference caused by the different exposure times. Using the proposed method, the RGB full-color image can be obtained while maintaining the high-sensitivity characteristic of the W pixels.

## 1. Introduction

A color filter array (CFA) performs an important role in the perception of color scenes for a single electrical image sensor [1,2]. However, owing to the fact that color filters only allow light within a specific spectral range to pass through, the light that arrives on the photodiode (PD) is attenuated by the CFA. For example, the Bayer pattern CFA [3] image sensor accepts visible light of only limited bandwidth range for each pixel. Although this approach is suitable for the color representation of the human visual system, it also limits the dynamic range capability of the image sensor.

**Figure 1:**
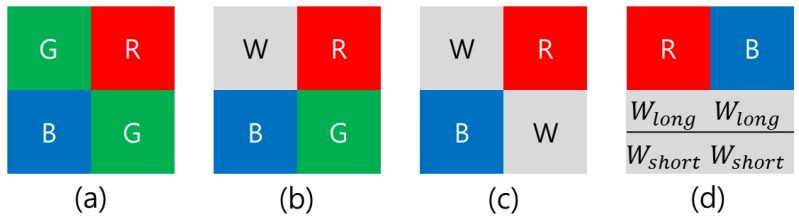
Examples of color filter array (CFA) patterns: (**a**) Bayer; (**b**) red–green–blue–white (RGBW); (**c**) red–white–blue (RWB); and (**d**) RWB with double-exposed W channel.

**Figure 2:**
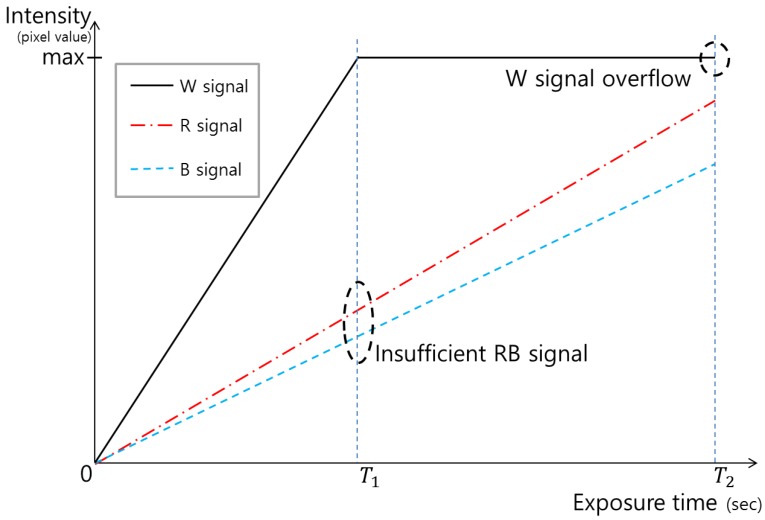
Intensity variation with exposure time for R, B, W signals with the RWB CFA image sensor.

In order to enhance the Bayer CFA sensitivity, a red–green–blue–white (RGBW) CFA image sensor was proposed [4,5,6,7]. This sensor obtains not only color information using the RGB color filters, but also luminance information using the white (W) filter. Because the RGBW CFA differs from the conventional Bayer CFA by only one pixel, it is highly efficient for the implementation of an Image Processing Chain (IPC). The insertion of the W filter renders the RGBW CFA more transparent than the Bayer CFA, and improves the image quality at poor illumination with minimal impact on color reproduction. However, the spatial resolution after color interpolation does not attain the level of the Bayer pattern CFA, because the RGBW CFA sensor is composed of many color components, as shown in Figure 1b. To overcome the spatial resolution problem while maintaining high-sensitivity imaging, Komatsu et al. [8] proposed an alternative approach. They developed an RWB pattern consisting of a repeating cell which is composed of two-by-two image pixels, with the two W pixels diagonally opposite from one another, and the other corners being R and B, as shown in Figure 1c. By replacing the G pixels in the RGBW pattern with W pixels, the conventional color interpolation method used for the Bayer CFA can be applied. Thus, the spatial resolution performance of the RWB CFA becomes comparable to that of the Bayer CFA. Recently, some industries have researched the RWB CFA pattern with interest, owing to the advantages of high resolution and high sensitivity. Further, this CFA pattern can be produced with minimal manufacturing cost, because the pattern is similar to the Bayer CFA widely used today [9]. However, owing to the high-sensitivity of the W pixels, these pixel values become rapidly over-saturated before the values of the pixels with the RB color filters reach an appropriate level, as shown in Figure 2. For overexposed conditions, the RWB pattern does suffer a loss of spatial resolution, as 50% of the array is comprised of luminance response photosites [7]. Moreover, although the spatial resolution is improved with the RWB CFA using W rather than G pixels, it is difficult to guarantee color fidelity because the color information cannot be accurately produced, owing to the lack of G pixels.

**Figure 3:**
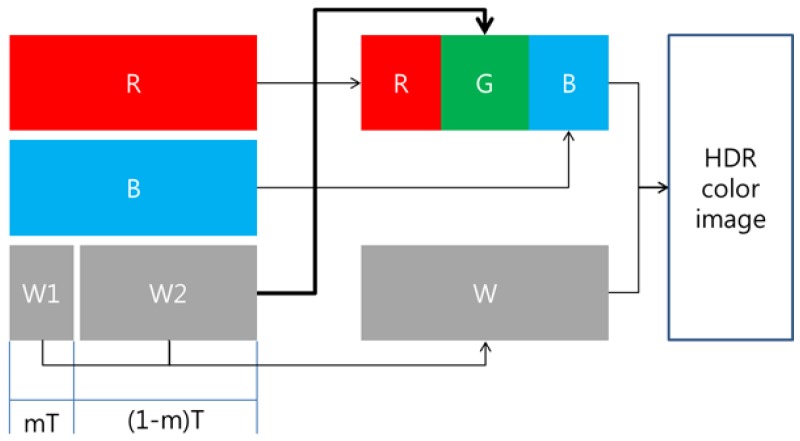
Block diagram of image acquisition system with RWB CFA image sensor. The bold line indicates proposed G channel restoration process. HDR: high-dynamic-range.

Two techniques are required to maximize the advantages of the RWB CFA. First, the G-channel information should be restored based on the correlation between W, R, and B. Second, the image should be fused, for which a high-dynamic-range (HDR) reconstruction technique that combines W information with RGB information is required (as shown in Figure 3). If images with restored G-channel information are obtained through the RWB CFA, improved spatial resolution can be realized on par with the resolution obtained through the RGBW CFA. However, as noted above, W pixels are saturated faster than R and B pixels, because W is more sensitive than R and B. This rapid saturation of W occurs when W is obtained along with R and B, as is the case for the existing Bayer CFA. Further, if W is saturated, G cannot be estimated accurately. To avoid W saturation, the image should be captured using a shorter exposure time. However, when the shutter speed is set according to the W exposure, the amount of light that reaches the RB pixels is reduced compared to the corresponding value for the Bayer CFA image sensor. Hence, other problems arise, such as a low signal-to-noise ratio (SNR) for R and B. To solve this issue, Park et al. [10] proposed a new RWB CFA pattern (see Figure 1d) which allows the pattern to obtain two W values at different exposure times. Despite, the loss of spatial resolution in the horizontal direction, the R and B pixels are arranged in odd rows and the W pixels are placed in even rows, so as to implement a Complementary Metal Oxide Semi-conductor (CMOS) image sensor-based readout method. A dual sampling technique is applied to the even rows, whereby two values with different exposure times can be obtained by scanning each row without modifying the readout method by which sensor data is acquired [11].

**Figure 4:**
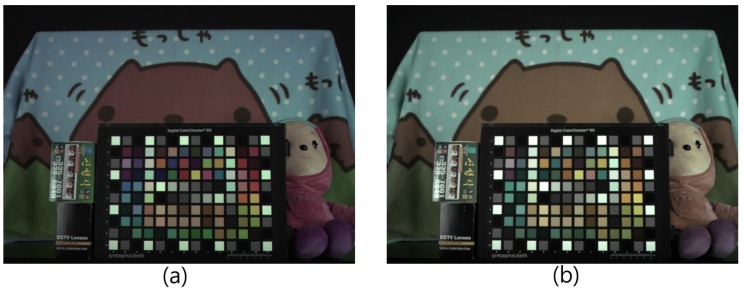
Color difference by use of W pixel (incandescent lamp): (**a**) Conventional Bayer CFA image; (**b**) Image obtained by RWB CFA image sensor.

By implementing the row-readout process and utilizing a dual sampling technique, it is possible to resolve the problem of W saturation and improve the sensitivity by simultaneously considering two W values. The bright portions of the image are best viewed through the short-integration-time column-parallel signal chain, and the darker portions of the image are best viewed through the long-integration-time column-parallel signal chain. The obtained R and B values exhibit a high SNR, because they are captured with the optimal exposure time. The disadvantage of the RWB CFA is the degradation of the spatial resolution in the horizontal direction (see Figure 1d). To overcome this weakness, Song et al. [12] proposed a Color Interpolation (CI) method that reduces the loss of spatial resolution in the horizontal direction for RWB patterns. The new CI method offers a better sensitivity while maintaining a high spatial resolution. However, with regard to color, mixing of G, R, and B color signals at the W pixel can result in extreme color degradation, as shown in Figure 4.

In this paper, we propose a G color restoration algorithm for an RWB CFA image sensor with a double-exposed W channel. In the proposed algorithm, two sampled W channels captured with different exposure times are fused into a single G-channel using the spectral correlation between the R, B, and W. Given the fact that the spectral response of the W pixel covers the complete range of visible-light, the color conversion process must consider the channel correlation between W and RB in order to accurately restore the G color information. With the G color being restored using the W pixels, it is possible to obtain an HDR image with a full RGB color range, as shown in Figure 3.

This paper is organized as follows: in Section 2, we present the problem statement for acquiring a color image using the RWB CFA image sensor; in Section 3, we discuss the problem of exposure-time selection; in Section 4, we propose the G-channel restoration method with a double-exposed W channel. In Section 5 and Section 6, we present the results of evaluation experiments and compare our solution with other state-of-the-art methods.

## 2. Color Degradation

To analyze the change in the chromaticity feature induced by the introduction of W pixel, the RGB color space was converted to the hue, saturation, and intensity (HSI) color space, according to

(1) $$\begin{array}{ccc} H & = & \cos\left(\frac{1}{2}\frac{\left(R-G)+(R-B\right)}{\sqrt{{\left(R-G\right)}^{2}+\left(R-B\right)\left(G-B\right)}}\right),\\ S & = & \frac{I-min(R,G,B)}{I}\;\;where\;a=min\left(R,G,B\right),\\ I & = & \frac{R+G+B}{3}, \end{array}$$

where min(·) represents the minimum of the three values. By replacing G pixels with W pixels, the intensity $I_{RWB}$ gets expressed as

(2) $$I_{RWB}=\frac{R+W+B}{3}.$$

Given that the intensity of G-pixels is always less than that of the W pixels, $I_{RWB}$ is increased, yielding a brighter image. With regard to the color, the saturation *S* decreases because the increase in the denominator is larger than that in the numerator in the term present on the right side of Equation (1).

(3) $$H=\cos\left(\frac{1}{2}\frac{\left(R-G)+(R-B\right)}{\sqrt{{\left(R-G\right)}^{2}+\left(R-B\right)\left(G-B\right)}}\right).$$

Further, hue *H* value changes are determined by the (R−W), ${\left(R-W\right)}^{2}$, and (W−B) terms in Equation (3). In general, the (R−W) term in the numerator decreases while the ${\left(R-W\right)}^{2}$ and (W−B) terms in the denominator increase. This implies that the *H* angle will converge to a specific point. Thus, a bright, low-saturated, and color-shifted image is produced by the RWB image sensor. Figure 4 illustrates the color degradation caused by the use of a W pixel instead of a G pixel. Although both Figure 4a,b are W balanced, the color fidelity of the RWB image (Figure 4b) shows that it is low-saturated and color-shifted.

## 3. Exposure-Time Selection Trade-Off

**Figure 5:**
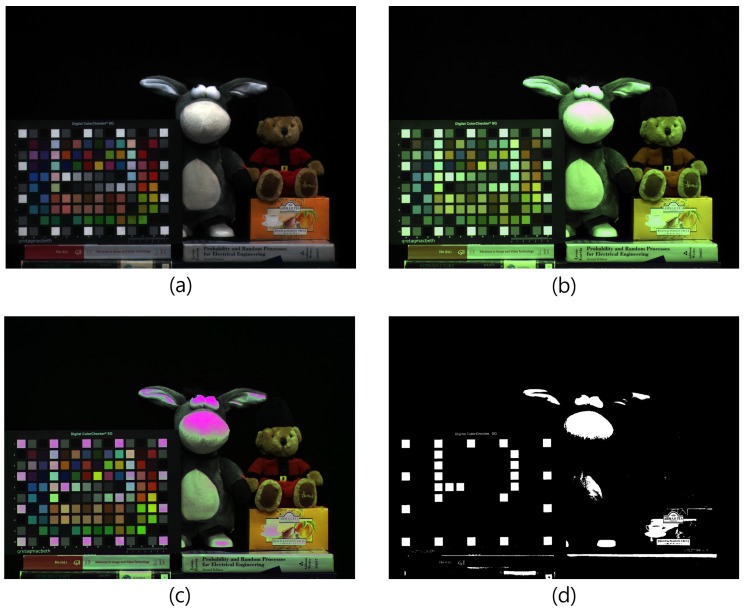
Color correction error due to the early saturation problem (fluorescent lamp): (**a**) Conventional Bayer CFA image; (**b**) Image obtained by RWB CFA sensor with saturated W pixels; (**c**) Color-corrected image of (b); and (**d**) Saturated area in W image.

The RWB CFA provides higher sensitivity and a higher SNR with increased light-conveyed image information compared to the Bayer CFA. However, recall that the W-pixel saturates earlier than the R and B pixel, as shown in Figure 2. The information loss caused by the saturated W reduces the color fidelity after the RGB color conversion process. Figure 5 shows an incorrect result from RWB-to-RGB color conversion caused by saturated W. In accordance with the explanation given in Section 2, Figure 5b shows a bright, de-saturated, and green-cast color. Given that the W pixels exhibit high overflow, the color degradation of the image is considerably more than that seen in Figure 4b.

Suppose that a high contrast image is obtained. When the exposure time is set according to the dark area of the image, a white hole will be observed in the bright area. In this scenario, the W pixel can no longer accept signals. This early saturation problem induces a loss of dynamic range in the bright areas. Although an advantage of introducing the W-pixel is the resultant high sensitivity, the details of the bright areas in the image cannot be expressed correctly when W is applied to generate an HDR image. Furthermore, if the shutter speed is set according to the W exposure, the amount of light that reaches the R and B pixels is reduced when compared with that of the Bayer CFA image sensor. This problem of an insufficient color signal is shown in Figure 2, where the exposure time is set according to $T_{1}$.

Moreover, the lack of an RB color signal decreases the SNR, which corresponds to an increase in the noise level and a decrease in the color fidelity of the generated color signal. Moreover, converting RWB to RGB through the use of a color correction matrix (CCM) results in the false color problem [13,14].

**Figure 6:**
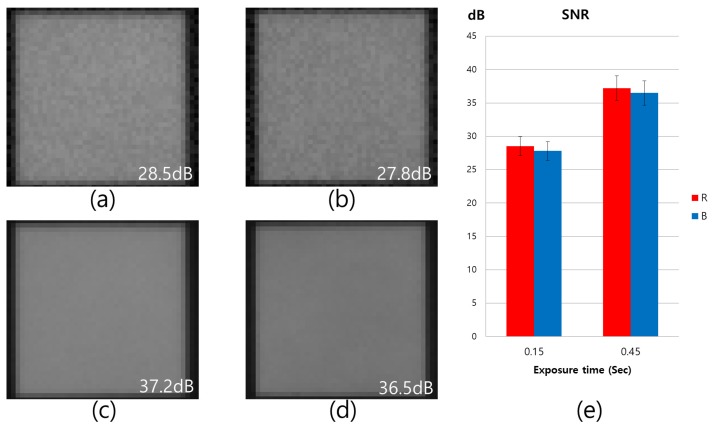
Noise level comparison between different exposure time: (**a**) Red channel (0.15 s); (**b**) Blue channel (0.15 s); (**c**) Red channel (0.45 s); (**d**) Blue channel (0.45 s); and (**e**) signal-to-noise ratio (SNR) versus exposure time.

In order to verify the color correction problem for the RWB CFA image sensor, we applied a CCM to Figure 5b. As a result, the color in the saturated area indicated in Figure 5d was not restored to the original color. In addition, degeneration to a purple color occurred. Therefore, the RWB CFA must set the exposure time relative to the W-pixel saturation point. Using this approach, however, an image with a low SNR is obtained because of the lack of R and B signals. Figure 6 shows a noise-level comparison for each color channel with different exposure times. The SNR level is indicated in the lower right portion of each image, as shown in Figure 6. Traditionally, SNR has been defined as the ratio of the average signal value $\mu_{sig}$ to the standard deviation $\sigma_{bg}$ of the background:

(4) $$SNR=20log_{10}\frac{\mu_{sig}}{\sigma_{bg}}dB.$$

For the Bayer CFA, the exposure time is generally set in accordance with the values given for Figure 6c,d. However, for the RWB CFA, a shorter exposure time must be set in order to prevent W-pixel saturation. In this scenario, a noise boost in each color channel is inevitable, as shown in Figure 6a,b. Comparing Figure 6a,c, it can be observed that the SNR level of R channel in Figure 6a is 8.7 dB, which is lower than that of Figure 6c. In case of the Figure 6a,b, the problem of the reduction in the amount of light that reaches the R and B pixels due to the shutter speed is set according to the W exposure.

To resolve the problem of early W saturation and improve the sensitivity by simultaneously using two W values, Park et al. [10] proposed a modified RWB CFA pattern using R, B, and double-exposed (rather than single-exposed) W. This scheme adopts a dual sampling technique [11] for the W pixels by applying a horizontal arrangement. While using a traditional CMOS Image Sensor (CIS) [15], a particular row is selected for readout. The sensor data from the selected row (for all columns) are simultaneously copied into a sampling capacitor bank at the bottom of the columns. The pixels in the row are then reset, read for a second time, and a new integration sequence is then initiated. This scan completes the readout for the selected row. The next row is then selected, and the procedure is repeated. In the dual-sampling architecture, two values with different exposure times can be obtained without modifying the readout method, which acquires the sensor data by scanning each row [11]. Using the dual sampling approach, it is possible to resolve the saturation problem for W. In addition, high SNR can be obtained for R and B.

Despite the advantage of the RWB image sensor scheme with double-exposed W, the differences in integration time between the RB and W can cause color degradation during the RWB-to-RGB color conversion process. In [10], RWB was converted to RGB using a color conversion process that simply multiplies W by a constant to obtain G. However, while the R and B pixels integrate the photocurrent within a single exposure time, the W-pixel integration time is divided into two exposure phases. Therefore, the double-exposed W-pixel value must be balanced in order to accurately convert the RWB values to RGB values.

## 4. G-Channel Restoration Using Double-Exposed W Channel

The color image produced by a CIS can be modeled as a spectral combination of three major components: the illuminant spectrum E(*λ*), the sensor function $R^{\left(k\right)}\left(\lambda \right)$, and the surface spectrum S(*λ*). The linearized camera response model for channel *k*, $C^{\left(k\right)}$, is defined as [16]

(5) $$C^{\left(k\right)}=\int_{w}E\left(\lambda \right)R^{\left(k\right)}\left(\lambda \right)S\left(\lambda \right)d\lambda ,$$

where *w* represents the spectral range of the visible band between 400 nm and 700 nm. Owing to the fact that the W pixel includes the RGB spectral band, the spectral information of the R and B bands must be eliminated in order to accurately extract the G-channel information from the W-pixel values. A straightforward method for mapping the RWB values to RGB values involves the use of a linear transformation in the form of a 3 × 3 matrix M, satisfying [14]

(6) Y=MX,

where Y is a 3 × *n* matrix of the RGB color vector under a canonical illuminant and X is a 3 × *n* matrix of the observed color vector of the RWB sensor responses (preferably under the same illuminant). The natural number *n* is the number of color samples. As a training set for determining the linear transfer motion coefficient, we used 96 standard colors of the GretagMacbeth ColorChecker SG, because these color samples are widely distributed. The transformation M is obtained by solving the minimization function

(7) $$\hat{M}=argmin_{M}{∥Y-MX∥}^{2}.$$

The linear transformation matrix can be derived using the pseudo inverse of matrix X, where

(8) $$\hat{M}=YX^{T}{\left(XX^{T}\right)}^{-1}.$$

The error calculating $\hat{M}$ from Equation (7) is minimized if the sensitivities of the RWB sensors are a linear transformation of the RGB color-matching functions [17]. Based on the spectral characteristics of the W pixels, we can assume that the value of the W pixel is a linear combination of the RGB pixel values. Thus,

(9) G=α(W)−β(R+B)+γ,

where *α* and *β* are the linear transformation coefficients obtained from Equation (8), and *γ* represents the offset value. The pseudo multiple capture scheme in [10] is applied at different exposure intervals to prevent early saturation of the W pixels. While the R and B pixels integrate the photocurrent during the integration time *T*, the W-pixel integration time is divided into two intervals: *mT* and (1-*m*)*T*, where (0 <*m*< 0.5). Therefore, it is imperative to balance the W-pixel value with the exposure time *mT*. Hence, we obtain

**Figure 7:**
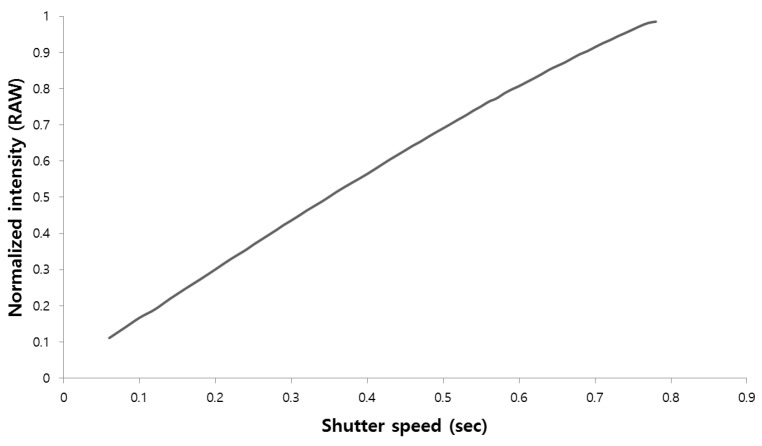
Camera response function of RAW image in W pixel.

(10) G=καW−β(R+B)+γ,

where *κ* represents the intensity balance coefficient determined by the gradient of the camera response function (CRF). As shown in Figure 7, the CRF of the raw image has linear characteristics with respect to the exposure time [18]. Because the dual sampling approach offers an advantage in terms of preserving signal linearity [11], the W-pixel values after the instant *mT* are *m* times those after the instant *T*. The G-channel information can be restored from W-pixel values using this linear response characteristic, even if the RB and W exposure times differ. By restoring the RGB color information based on the G-channel restoration, it is possible to create an HDR image using the W channels with two different exposure values. In this paper, the outputs after exposure times of *mT* and (1-*m*)*T* are denoted by $W_{s}$ and $W_{L}$, respectively. If $W_{L}$ is saturated, the proposed method restores the G channel using $W_{s}$, which has a low exposure value.

(11) $$\begin{array}{c} G=\left\{\begin{array}{cc} \kappa_{mT}\alpha W_{s}-\beta \left(R+B\right)+\gamma , & W_{L}>saturated,\\ \kappa_{(1-m)T}\alpha W_{L}-\beta \left(R+B\right)+\gamma , & else, \end{array}\right. \end{array}$$

where $\kappa_{mT}$ and $\kappa_{(1-m)T}$ represent the intensity balance coefficient for the exposure times of mT and (1−m)T, respectively. From Equation (11), the G-pixel values are generated using $W_{s}$ and $W_{L}$, depending on the presence of the saturation region in the image produced with $W_{L}$.

## 5. Experimental Results

The proposed method was tested using images captured under the following standard illuminations: sunlight, incandescent lamp, and fluorescent lamp. Since the spectra of these light sources were spread over a wide range, we used the illumination values produced by these light sources to determine the target illuminance values. Table 1 shows the illumination settings of the test images in this paper.

We have used 96 standard colors of the GretagMacbeth ColorChecker SG as training set in order to compute the correlation coefficients. These colors were included in the training set because they are widely distributed in the color space. The input RWB and target RGB images were obtained by using a camera system with internal color filter wheels. The 96 patches were manually segmented, and we used the average RGB values of each patch. The resulting average RGB values in the input image and reference image were used to derive a set of color transformation models, as presented in Equation (8). Moreover, we also measured the tristimulus values of each of the 96 patches using a spectrophotometer.

To evaluate the performance achieved by adopting double-exposure with the RWB camera system, a saturated image with high contrast was tested through the application of the color correction method.

**Figure 8:**
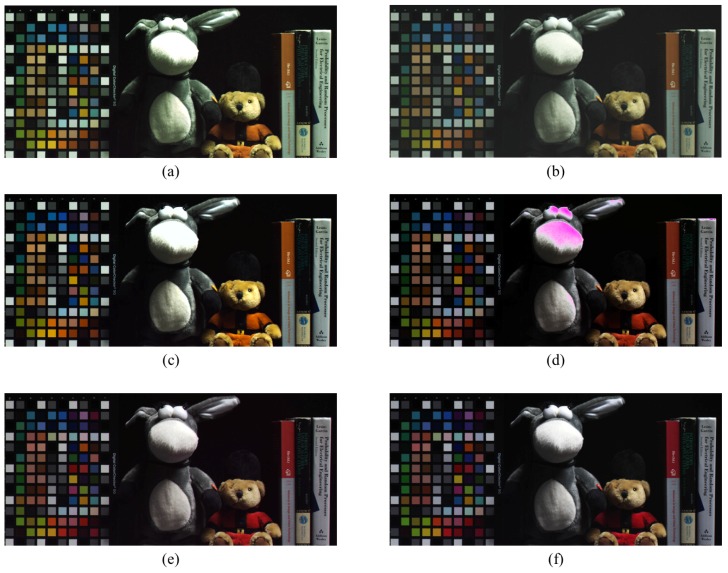
Experimental results (fluorescent lamp): (**a**) RWB image before RGB color conversion without double-exposed W; (**b**) RWB image before RGB color conversion with double-exposed W; (**c**) Color correction of (a) by color transfer; (**d**) Color correction of (a) by applying CCM; (**e**) RGB color image (target image); and (**f**) Proposed method with (b).

Figure 8 shows a comparison between an RWB input image and the results of various methods. Figure 8a shows the G-cast image discussed in Section 2. The white area caused by the high-sensitivity of the W pixel is apparent. On the other hand, Figure 8b shows the image obtained with a double-exposed W channel. In this case, the merged $W_{s}$ and $W_{L}$ demonstrate that the dynamic range of the bright area is preserved. A conventional color correction method was tested on the image used in Figure 8a, and the result is shown in Figure 8c. In this image, the color correction result obtained using the approach described in [19] is shown. This method manipulates colors in the color space and restores them to the target colors. Thus, the intensity of (a) is preserved, and the color deviation is improved slightly. However, the use of color transfer in the color domain does not improve the image at all. This result implies that the mixed color information contained in the W pixels—which must be eliminated in order to restore the G color—is retained in Figure 8c. Moreover, the saturated W pixels still exist because of the early saturation problem. Figure 8d shows the color correction result obtained by the application of the RWB-to-RGB CCM. The CCM extracts G-color information from the W pixels by subtracting R and B from W. Nevertheless, when the W pixels are saturated, the R and B subtraction causes the “lack of G” problem. The purple-colored area in Figure 8d indicates the occurrence of this false color restoration problem. Finally, when the proposed method is employed (see Figure 8f), the color fidelity of the resultant image outperforms that obtained using the previous color correction method, without the appearance of the early saturation problem. The saturation problem is overcome using the dual shutter scheme, and the G color information is restored by subtracting R and B from W while considering the energy balance between the different exposure times. Another experimental dataset was also tested in order to compare the color fidelity more closely.

**Figure 9:**
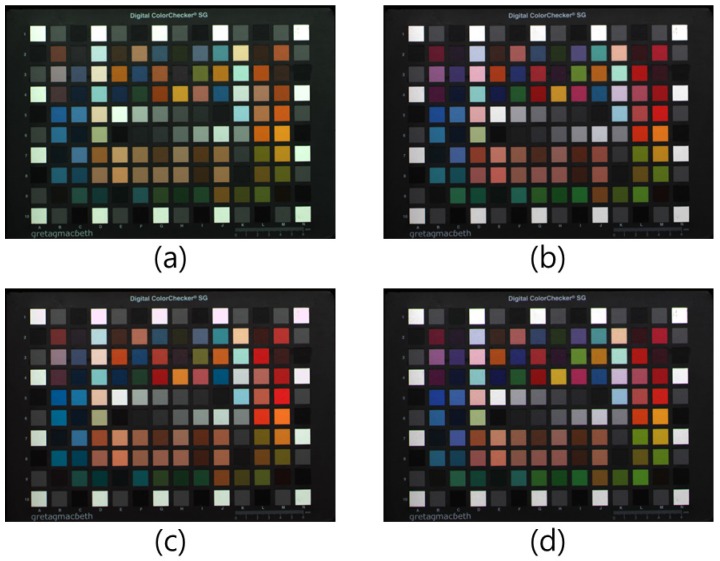
Experimental results (fluorescent lamp): (**a**) RWB image before RGB color conversion; (**b**) RGB color image; (**c**) 3 × 3 color correction matrix (CCM); and (**d**) Proposed method.

Figure 9 shows the experimental results under a fluorescent lamp with 300 lx illumination with 5000 K color temperature. In our experiment, we measured the illumination level using an illuminometer. Given the fact that the G channel in Figure 9a is replaced by a W channel, the overall colors of the image are low-saturated and green-cast after the white balance. In Figure 9b,d, an RGB full-color image and G-channel-restored image are shown, respectively. In Figure 9c, result with the conventional color correction method is shown. Comparing Figure 9b,c, the H and S of the light green on the bottom right side of Figure 9c are not restored correctly. In contrast, comparing Figure 9b,d, the overall colors of each patch are well restored. This implies that the proposed method successfully restores the G color component from the W-pixel information.

**Figure 10:**
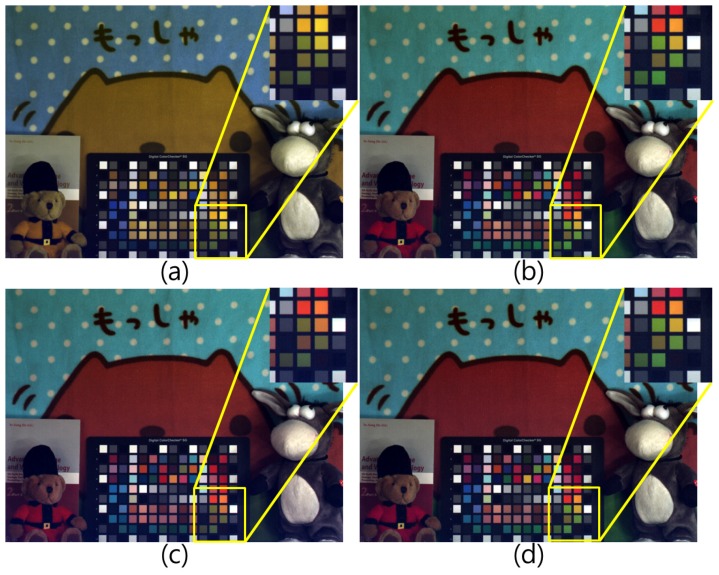
Experimental results (incandescent lamp): (**a**) RWB image before RGB color conversion; (**b**) RGB color image; (**c**) 3 × 3 CCM; and (**d**) Proposed method.

Another set was tested under an incandescent lamp with 200 lx illumination with 3000 K color temperature. Figure 10a represents an RWB image obtained under the incandescent lamp. The color channels were white balanced without considering the color degradation caused by the lack of the G channel. Therefore, the overall colors of the image are different when compared to Figure 10b. The comparison of Figure 10c,d shows that the overall colors of each color patch and object were similar to the RGB target image (Figure 10b). However, the green color patches—which enlarged with the yellow box—were different. Figure 10d is much more similar to the target image in Figure 10b.

**Figure 11:**
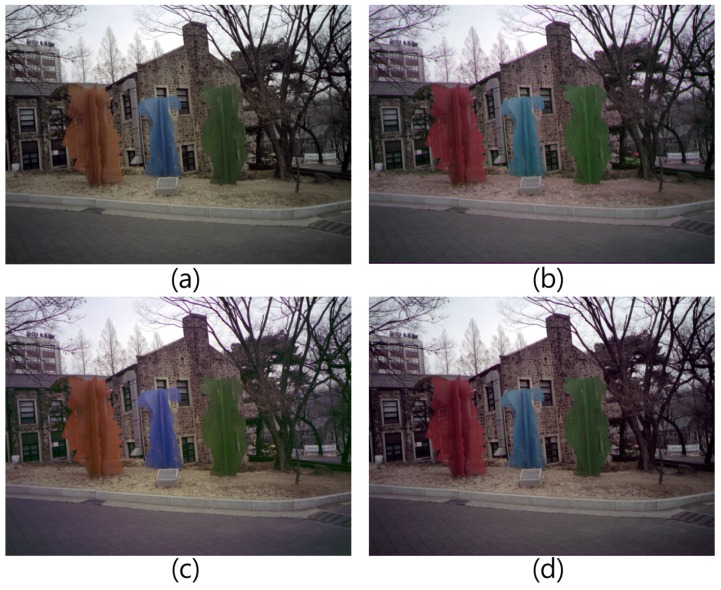
Experimental results (sunlight): (**a**) RWB image before RGB color conversion; (**b**) RGB color image; (**c**) 3 × 3 CCM; and (**d**) Proposed method.

Figure 11 shows the experimental results under sunlight. When comparing Figure 11c,d, the resulting image of the proposed method restored the distorted color well, especially the red color of the sculpture.

As an error criterion, the angular error was calculated. Considering the total number of *L* color sample entities in the training set, the angular error for the *l*-th color was defined as

(12) $$\theta_{l}=\cos^{-1}\left(\frac{a_{l}\cdot b_{l}}{|a_{l}\left|\cdot \right|b_{l}|}\right),$$

where $\theta_{l}$ is the angular error between the target color vector $a_{l}$ and the color restoration result $b_{l}$. Here, the “·” represents the inner product of two vectors and |a| represents the magnitude of a. In addition, we measured the color difference ΔE of each color sample in the CIELAB color space defined by

(13) $$\Delta E=\sqrt{{\left(\Delta L^{*}\right)}^{2}+{\left(\Delta a^{*}\right)}^{2}+{\left(\Delta b^{*}\right)}^{2}}.$$

We regarded the average of ΔE as the color correction error.

**Figure 12:**
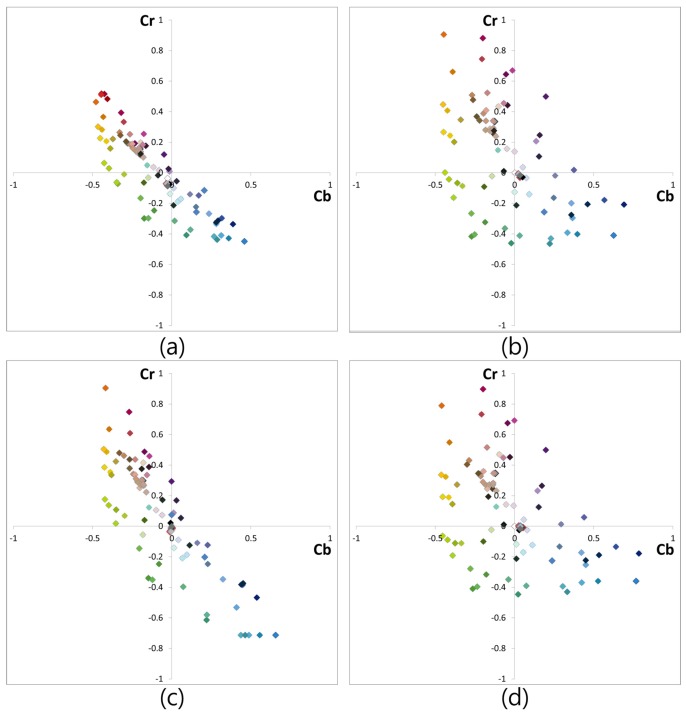
Color distribution of 96 color patches of the GretagMacbeth ColorChecker SG: (**a**) RWB image before RGB color conversion; (**b**) RGB color image; (**c**) 3 × 3 CCM; and (**d**) Proposed method.

Figure 12 shows the color distribution of all 96 color patches of the GretagMacbeth ColorChecker SG of each image of Figure 9. Each color patch value was calculated in the normalized YCbCr domain. For the gamut representation independent of the luminance component, normalized YCbCr domain was used. As shown in Figure 12a, each color component is gathered along the red and blue direction. This implies that there is a lack of G information in the RWB image. When Figure 12b,c are compared, it is evident that the color distribution of the conventional color correction method is not efficient. The conventional color correction matrix manipulates color to red and blue directions well. However, in the direction of green (quadrants 1 and 3), the color components were not spread out. That is the reason why the color of Figure 10c shows low saturation in the green channel. In contrast, the color distributions of Figure 12b,d are very similar. Owing to the successful restoration of G color information from the W channel as per the proposed method, color distribution along the direction of quadrants 1 and 3 was seen to be spread out well.

**Figure 13:**
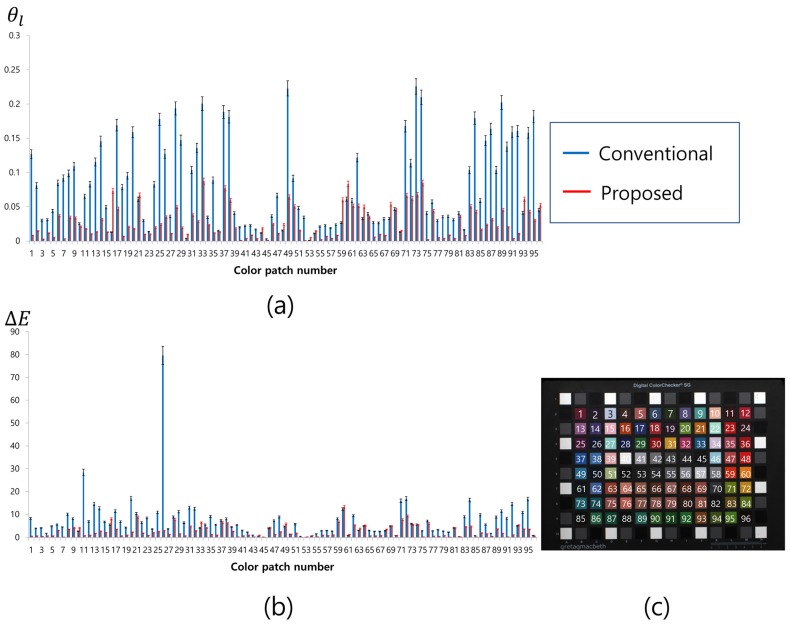
Performance measurement graph for color patches of GretagMacbeth ColorChecker SG. The red bar indicates performance of proposed method: (**a**) Angular error; (**b**) Delta E; and (**c**) Numbering of patches.

Figure 13 and Table 2 show the average $\theta_{l}$ of the standard color chart patches and ΔE, respectively. A performance evaluation of the proposed and conventional methods for various colors in the color chart was conducted by comparing the results of these methods for the RGB target image shown in Figure 9b. The performance of the proposed method for each color patch is indicated by the red bar in Figure 13, whereas the blue bar represents the performance of the conventional method. The error bars at each point represent the certain confidence interval. Since the color of the RWB image was severely distorted, the conventional color correction method yields lower color fidelity compared to the proposed method, as is evident from Figure 13. Especially, a difference of $\theta_{l}$ and ΔE is noticeable in the green and purple color groups. As mentioned above, the color distribution of the result of the conventional method was not well-spread in the direction of green and purple (quadrants 1 and 3), as shown in Figure 12. This implies that the G channel has not been restored correctly.

**Figure 14:**
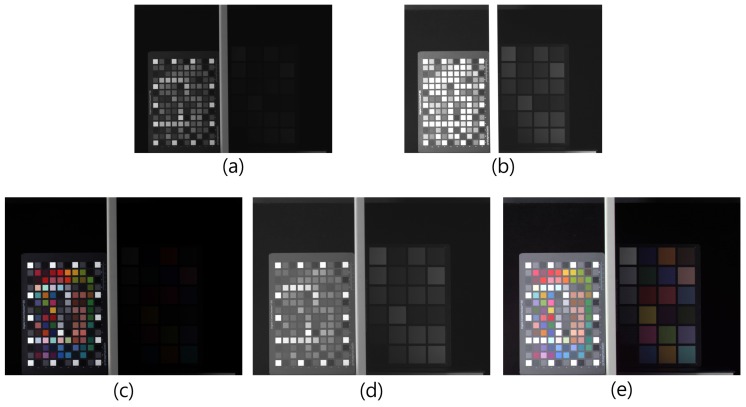
HDR image generation process (fluorescent lamp): (**a**) $W_{s}$ image with shutter speed (0.1 s); (**b**) $W_{L}$ image with shutter speed (0.1 s); (**c**) Color-restored image with proposed method; (**d**) Merged luminance image by linear averaging (a) and (b); and (**e**) HDR image by using (c) and (d).

Moreover, because of the double-exposed white channel that prevents early saturation of the W pixel, the exposure level must be balanced in order to restore the G channel from the W, R, and B information, as shown in Equation (11). The proposed method restores the G channel information from the W channel by considering the problems mentioned above. Table 2 lists the average of the $\theta_{l}$ and ΔE values obtained from the values for Figure 9b–d. The values measured for the proposed method outperform those of the other method. As a result, it is possible to implement HDR imaging using the RWB image sensor with the proposed method. Figure 14 shows a series of images in the process of creating an HDR image. The three images (Figure 14a,b and a luminance image of Figure 14c) were used to improve the HDR luminance $L_{hdr}$ sensitivity at pixel position (i,j). We have

(14) $$L_{hdr}={\hat{w}}_{s}^{hdr}\left(i,j\right){\hat{W}}_{s}\left(i,j\right)+{\hat{w}}_{L}^{hdr}\left(i,j\right){\hat{W}}_{L}\left(i,j\right)+{\hat{w}}_{lumi}^{hdr}\left(i,j\right)L\left(i,j\right),$$

where ${\hat{w}}_{s}^{hdr}$, ${\hat{w}}_{L}^{hdr}$, and ${\hat{w}}_{lumi}^{hdr}$ represent the normalized HDR weight values for interpolated short-(${\hat{W}}_{s}$) and long-exposed W (${\hat{W}}_{L}$), and the (*L*) value from the RGB values, respectively. These values are calculated from

(15) $${\hat{w}}_{k}^{hdr}\left(i,j\right)={\left[\sum_{k}w_{s}^{hdr}\left(i,j\right)\right]}^{-1}w_{k}^{hdr}\left(i,j\right),$$

where $k=\left\{{\hat{W}}_{k},{\hat{W}}_{L},L\right\}$. The $w_{k}^{hdr}$ for each image are calculated from

(16) $$w_{k}^{hdr}\left(i,j\right)=C_{k}^{\alpha }\times S_{k}^{\beta }\times E_{k}^{\gamma },$$

where $C_{k}$, $S_{k}$, and $E_{k}$ represent the quality value measures for the contrast, saturation, and well-exposedness, respectively; *α*, *β*, and *γ* are the respective weight values of each measure. A detailed description of these quality measures is presented in [20]. Figure 14c shows the color-restored image that has been obtained by applying the proposed method. Figure 14d shows an HDR luminance channel, constructed using Figure 14a,b. By Figure 14c,d, an HDR image is generated (Figure 14e), having rich color information and the advantage of high sensitivity gain. Thus, using the proposed method, the usability of the RWB image sensor is further improved.

**Figure 15:**
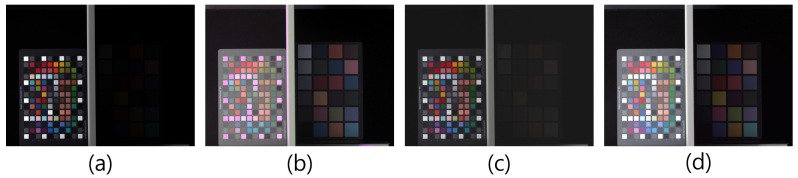
Comparison of HDR reconstruction results: (**a**) Bayer CFA; (**b**) RWB CFA with saturated W; (**c**) RWB CFA with unsaturated W; and (**d**) double-exposed RWB CFA.

Figure 15 shows the comparison results for a test image captured using the RWB CFA pattern. The test image includes both a bright region and a dark region. The average brightness of Figure 15c was lower than that recorded in the other results, due to the shorter exposure time used to prevent the saturation of W. If W is saturated, false color information is restored due to the inaccurate estimation of the G-channel, as shown in Figure 15b. The SNR of each image in Figure 15 is compared in Table 3. According to the SNR values in Table 3, the CFA patterns with W-pixel recorded larger values compared to those with the Bayer CFA pattern. For the RWB CFA with unsaturated W, the SNR value is smaller than that of the other CFA with W, owing to the shorter exposure time which avoids the saturation of W. Although the SNR value of the dark side is higher than that of the Bayer CFA, the usage of W-pixels has no advantage because of the low SNR in the bright side. In contrast, for the RWB CFA with saturated W, the SNR value in the dark side is higher than that of the other CFA with W. In this case, the amount of light that reaches the R and B pixels reaches an appropriate level. However, the saturated W reduces color fidelity in the bright side after the RGB color conversion process. Due to the distorted color information, the SNR value in the bright side cannot record the highest value. On the other hand, the double-exposed RWB CFA pattern recorded a larger value than the others in the bright side. Furthermore, the color information in the bright side is preserved because of the prevention of early saturation phenomenon by the double-exposed W. Using the RWB CFA with double-exposed W, the RGB full-color image can be obtained while maintaining the high-sensitivity characteristic of the W-pixel.

## 6. Conclusions

In this paper, a G-channel restoration algorithm for an RWB CFA image sensor with double-exposed W channel was proposed. Given that the RWB CFA image sensor does not have G pixels, W pixels are used to generate the G information. Because the missing “G” color information that is included in the W channel cannot be restored with a saturated W, multiple captures with dual sampling are necessary in order to solve this early W-pixel saturation problem. The proposed method restores G color information from the W channel by considering the energy difference caused by the different exposure times. The proposed algorithm outperformed conventional methods, which was confirmed by objective metrics and by a comparison using actual images. In future research, we intend to study image fusion algorithms, and apply this method to high resolution R, W, and B channels generated by using the proposed algorithm. Using this method, RGB full-color images can be obtained while maintaining the high-sensitivity of the W channel.

## Figures and Tables

**Table 1:** Illumination setting.

Light Source	Color Temperature		Related Figure
Fluorescent	5000 K	200 lx	Figure 5
		300 lx	Figure 8, Figure 9 and Figure 14
Incandescent	3000 K	150 lx	Figure 4
		200 lx	Figure 10
Sunlight	6000 K	340 lx	Figure 11

**Table 2:** Average angular error and ΔE.

Setup	Angular Error		Color Difference	
	3 × 3 CCM	Proposed Method	3 × 3 CCM	Proposed Method
Fluorescent lamp	0.736	0.273	7.148	2.614
Incandescent lamp	0.832	0.334	9.624	3.188
Sunlight	1.521	1.027	10.973	7.505

**Table 3:** SNR comparison of HDR reconstructed images.

Pattern’s Type	Bright Side	Dark Side
Bayer CFA	38.83 dB	16.73 dB
RWB CFA with saturated W	41.08 dB	25.18 dB
RWB CFA with ussaturated W	36.73 dB	21.38 dB
RWB CFA with double-exposed W	43.30 dB	23.27 dB
